# Mammary Gland Tropism and Milk-Mediated Transmission of H5N1 in Dairy Cattle: Implications for One Health Surveillance

**DOI:** 10.3390/vetsci13090869

**Published:** 2026-08-26

**Authors:** Kehui Zhang, Yixiang Wang, Xuanrong Wang, Fang Wang, Guanlong Xu, Jie Wang, Zhaofei Wang, Yuqiang Cheng, Heng’an Wang, Yaxian Yan, Jianhe Sun, Jingjiao Ma

**Affiliations:** 1 Shanghai Key Laboratory of Veterinary Biotechnology, Key Laboratory of Urban Agriculture (South), Ministry of Agriculture, School of Agriculture and Biology, Shanghai Jiao Tong University, Shanghai 200240, China; zkh4243@sjtu.edu.cn (K.Z.);; 2 China Institute of Veterinary Drug Control, Beijing 100081, China

**Keywords:** dairy cattle, mammary gland tropism, viral shedding in milk, cross-species transmission, One Health

## Abstract

Highly pathogenic avian influenza H5N1 virus (H5N1 HPAIV) has long circulated in wild waterfowl and poultry and continues to cross species barriers, posing sustained threats to livestock productivity and public health. The 2024 outbreak of H5N1 HPAIV in dairy cattle across multiple U.S. states represents the first recognized large-scale transmission event of this virus in ruminants, reshaping our understanding of its host range and transmission ecology. This review summarizes recent advances in the epidemiology, virological characteristics, transmission, pathogenesis, surveillance, and control of dairy cattle-associated H5N1. The initial multistate outbreak was dominated by clade 2.3.4.4b genotype B3.13, whereas subsequent independent introductions of genotype D1.1 demonstrated that repeated avian-to-cattle spillover also contributes to the evolving outbreak ecology. A defining feature is efficient replication in bovine mammary epithelial cells, resulting in high viral titers in milk, supporting milk-associated exposure and milking-related contamination as plausible components of transmission, although the relative contribution of different routes remains unresolved. Infected cattle typically show reduced feed intake, a marked decline in milk production, abnormal milk, and mild systemic signs, whereas severe respiratory disease and mortality are uncommon. Mutations such as PB2-M631L, PA-K497R, and changes in NP and NS1 may promote replication and immune evasion in bovine cells, while genotype-specific PB2 adaptations highlight the potential for further mammalian adaptation. Human infections reported to date have been predominantly mild and associated with occupational exposure, with no evidence of sustained human-to-human transmission. Experimental vaccine studies have begun to demonstrate immunogenicity in cattle, although protection against mammary infection, viral shedding, and transmission remains to be established. Future priorities include clarifying mammary tropism and transmission dynamics, while strengthening diagnostics, farm biosecurity, vaccination, evaluating cattle vaccination strategies, and integrated One Health surveillance.

## Simple Summary

Since March 2024, H5N1 highly pathogenic avian influenza has been circulating among U.S. dairy cattle. Unlike the predominantly respiratory disease typically associated with influenza virus infection, this virus mainly infects the mammary gland and is shed in large quantities through milk, which can contaminate milking equipment and the farm environment. The infection leads to reduced milk yield, and the milk becomes thick and yellow. The virus transmission may occur via multiple routes, including milk contamination, milking equipment, and possibly air or wastewater on farms. However, the principal transmission pathway remains unclear. Notably, experimental evidence has shown that co-housed susceptible animals do not readily acquire infection, and intramammary inoculation of a single quarter does not consistently result in transmission to other quarters in the same animal, suggesting that within-herd transmission is likely multifactorial and cannot be attributed solely to milk-associated exposure. Farm workers who handle raw milk or sick cows are at risk, mostly through eye contact or breathing in virus particles. Control measures including isolation of sick cows, equipment disinfection, milk testing, and avoiding raw milk feeding to animals are necessary but challenging. Therefore, establishing an integrated One Health system is essential to effectively safeguard both animal and public health.

## 1. Global Epidemiology and Cross-Species Transmission of H5N1 HPAIV: A Historical Overview

Since its emergence, highly pathogenic avian influenza H5N1 virus (H5N1 HPAIV) has established sustained circulation in wild waterfowl and poultry, causing multiple avian outbreaks worldwide. In recent years, the sustained circulation of clade 2.3.4.4b H5N1 has markedly altered the transmission landscape of H5N1, making it a central concern in global avian influenza prevention and control. Unlike previous outbreaks that were largely confined to wild birds and poultry, the global animal outbreaks attributed to clade 2.3.4.4b H5N1 since 2020 have been marked by broader geographic spread, more frequent genetic reassortment, and more prominent mammalian infections [1]. A previous review reported that, between 2020 and 2023, natural H5N1 infections had been documented in more than 48 mammalian species across 26 countries worldwide [2,3,4]. Both the affected host range and geographic distribution have substantially exceeded those recorded during previous epidemic waves from 2003 to 2019, suggesting that the ecological niche of the virus is continuing to expand and that the risk of cross-species transmission is increasing accordingly [4].

Against this global epidemiological background, the 2024 H5N1 outbreak in U.S. dairy cattle constitutes a landmark event in the epidemiological history of H5N1 HPAIV [5]. Early field investigations showed that affected dairy cattle mainly exhibited reduced feed intake, decreased rumination, a sudden drop in milk production, and thick, yellow, colostrum-like milk, while overall mortality remained low [6]. The subsequent confirmation by the U.S. Department of Agriculture of H5N1 infections in dairy herds across multiple states marked the first recognized large-scale transmission of this virus in ruminants [7]. The initial multistate expansion was predominantly associated with clade 2.3.4.4b genotype B3.13; however, subsequent surveillance demonstrated that the epidemiology of H5N1 in dairy cattle was not restricted to this genotype. In early 2025, genetically distinct genotype D1.1 viruses were detected in dairy cattle in Nevada and Arizona, with whole-genome sequencing indicating separate introductions that were also distinct from the previously circulating B3.13 cattle lineage [8]. A further D1.1 virus detected in a Wisconsin dairy herd in December 2025 was phylogenetically distinct from the Nevada and Arizona viruses and closely related to locally circulating avian viruses, supporting another independent avian-to-cattle spillover event [9]. These findings indicate that the U.S. dairy-cattle H5N1 event comprises both sustained cattle-associated expansion of the dominant B3.13 lineage and recurrent introductions of genetically distinct H5N1 viruses from avian reservoirs.

Unlike previous sporadic H5N1 infections in wild or companion mammals, the dairy cattle outbreak occurred within a high-density dairy production system characterized by continuous production and frequent human–animal interfaces [5]. The resulting epidemiology is therefore shaped by processes operating at multiple levels, including recurrent exposure to viruses circulating in wild and domestic birds, movement of infected cattle between farms, potential milk- and milking-associated exposure, environmental contamination, farm biosecurity practices, and occupational contact. The independent D1.1 introductions detected after the initial B3.13 expansion further demonstrate that dairy cattle remain susceptible to H5N1 viruses circulating in avian reservoirs [8,9]. This evolving epidemiological pattern has established dairy farming systems as an important interface for investigating viral adaptation, cross-species transmission, and One Health risk [6,10].

Following the U.S. dairy cattle outbreak, a rapid expansion of studies has emerged, addressing critical questions on viral origin and genomic epidemiology, transmission routes within herds and between farms, viral shedding in milk and dairy product safety, adaptive evolution in mammalian hosts, cross-species transmission events, and farm-level testing, public health surveillance, and biosecurity measures. These studies have advanced our understanding of dairy cattle-associated H5N1 from multiple perspectives [6,10,11]. Experimental infection and pathological studies have shown that mammary gland tropism and high viral loads in milk are key features that distinguish H5N1 infection in dairy cattle from classical respiratory influenza. Studies on environmental stability and milk surveillance further suggest that raw milk, milking equipment, and the farm environment may act as sources of contamination and vehicles for mechanical transmission, with important implications for surveillance and early warning systems [6].

Although these studies have considerably advanced our knowledge of dairy cattle-associated H5N1, a comprehensive landscape of the outbreak and its underlying dynamics has yet to emerge. Genomic epidemiology can illuminate viral origins and spread, but cannot by itself determine the relative contributions of distinct transmission routes. Experimental infection studies can define mammary tropism, shedding kinetics in milk, and pathogenicity in animal models, but these findings require corroboration under natural exposure conditions. While public health surveillance has prioritized diagnostics, farm biosecurity, and product safety, these efforts are seldom integrated with evolutionary and epidemiological investigations of within-herd and between-farm spread [11]. Therefore, several key questions remain unresolved, including the relative importance of different transmission routes within cattle herds, the true scale of mild or subclinical infections, the rate of asymptomatic infection among occupationally exposed populations [10], whether sustained transmission in dairy cattle may further promote the accumulation of mammalian-adaptive mutations, and the effectiveness of current diagnostic and control strategies under real farm conditions [12,13].

Therefore, this review focuses on research advances since the 2024 outbreak of H5N1 in U.S. dairy cattle. It first outlines the detection, spread, and epidemiological characteristics of H5N1 in U.S. dairy cattle, and then summarizes the virological features, host adaptation, and evidence for cross-species transmission of dairy cattle-associated H5N1. It then discusses transmission routes within cattle herds, clinical and pathological manifestations, viral shedding in milk, and public health risks [13]. The review concludes with an appraisal of current surveillance, control, and risk assessment gaps, and proposes key research priorities under the One Health framework [12], synthesizing emerging consensus and persistent controversies to guide future investigations into transmission ecology, zoonotic risk, and intervention strategies.

### Literature Search Strategy

This narrative review was based primarily on the literature identified through PubMed, supplemented by official reports and surveillance updates from the U.S. Department of Agriculture (USDA), Animal and Plant Health Inspection Service (APHIS), Centers for Disease Control and Prevention (CDC), and Food and Drug Administration (FDA). The literature search was updated through 31 July 2026, with particular emphasis on studies published from March 2024 to July 2026, following the recognition of H5N1 infection in U.S. dairy cattle. Earlier studies were also included when they provided relevant background on influenza virus biology, bovine mammary susceptibility, receptor distribution, mastitis, or related mechanisms. Searches used combinations of terms including “H5N1,” “highly pathogenic avian influenza,” “HPAIV,” “influenza A virus,” “B3.13,” “D1.1,” “dairy cattle,” “dairy cows,” “bovine,” “mammary gland,” “mastitis,” “milk,” “milk shedding,” “mammary tropism,” “transmission,” “pathogenesis,” “sialic acid receptor,” “surveillance,” “vaccination,” and “occupational exposure.” Peer-reviewed English-language original research articles and relevant reviews addressing H5N1 infection in cattle, mammary pathogenesis, viral shedding, transmission, diagnostics, epidemiology, vaccination, and One Health implications were prioritized. Official agency reports were additionally used to verify outbreak chronology, genotype detections, surveillance activities, and public-health guidance. Titles and abstracts were screened for relevance, followed by full-text assessment of potentially eligible studies.

## 2. Outbreak and Epidemiological Characteristics of H5N1 in U.S. Dairy Cattle from 2024

The emergence of H5N1 in U.S. dairy cattle in 2024 was a landmark event that reshaped the epidemiological landscape of clade 2.3.4.4b H5N1 HPAIV [5]. As early as February 2024, a syndrome characterized primarily by abnormal milk production was observed on several dairy farms in Texas. Affected cattle showed reduced feed intake, decreased rumination, fever, depression, mild respiratory or gastrointestinal signs, along with a sudden decline in milk yield, with milk becoming thick, yellow, and colostrum-like in appearance. Field investigations indicated that morbidity reached approximately 10–15% on some farms, whereas mortality remained low. Outbreaks typically peaked 4–6 days after the first cases appeared and resolved within 10–14 days [6]. Because the early clinical signs were nonspecific and neither high mortality nor severe respiratory disease was a dominant feature, the outbreak was not immediately recognized as H5N1 HPAIV infection.

Subsequently, dairy herds in multiple U.S. states were reported with similar clinical signs, and H5N1 HPAIV was detected in milk, nasal swabs, and related tissue samples from affected cattle. In March 2024, the U.S. Department of Agriculture officially confirmed H5N1 clade 2.3.4.4b infection in dairy cattle, marking the first recognized large-scale transmission of H5N1 HPAIV in cattle or other ruminants worldwide [5]. Unlike previous sporadic H5N1 infections in mammals such as cats, foxes, sea lions, and mink, this outbreak occurred in a high-density dairy production system characterized by continuous milking operations and frequent human–animal contact, and it continued to spread across multiple dairy farms [2]. These features suggest that dairy cattle may have become a new mammalian host involved in the transmission of H5N1.

Epidemiological and genomic analyses indicate that the U.S. dairy cattle H5N1 outbreak is predominantly associated with clade 2.3.4.4b genotype B3.13 [4,14]. The dominant B3.13 lineage most likely arose from a single avian-to-cattle spillover in late 2023, followed by several months of cryptic circulation before recognition of the outbreak and subsequent sustained transmission within cattle populations and multistate dissemination [4,15]. Cattle movement and the highly interconnected U.S. dairy production network likely contributed to this geographic expansion, although the precise mechanisms of within- and between-herd transmission remain incompletely resolved. In addition to dairy cattle, infections or deaths in cats, wild birds, and raccoons were also observed on affected farms. Whole-genome sequencing showed that H5N1 viruses from these animals belonged to genotype B3.13 and were phylogenetically closely related to viruses detected in cattle on the same farms [5,6]. In particular, basal sequences from viruses detected in cats and raccoons were derived from cattle viruses, suggesting possible spillover from dairy cattle to cats and raccoons. The outbreak therefore involved a complex network of cross-species exposure and transmission within farm ecosystems, including dairy cattle, cats, wild birds, and peridomestic mammals [2,5].

Among human cases reported to date in association with dairy cattle H5N1, occupational exposure has been the predominant route, with conjunctivitis being the most common manifestation and some cases also presenting with upper respiratory symptoms [16]. Animal model studies suggest that the route of exposure influences infection outcomes: oral ingestion of contaminated milk generally results in limited or subclinical infection in non-human primates, whereas intranasal or intratracheal exposure is more likely to cause respiratory disease, with lower respiratory tract exposure leading to more severe pulmonary lesions [17]. Health monitoring and diagnostic testing should therefore be strengthened for dairy farm workers, veterinarians, milk handlers, and other exposed personnel, together with timely clinical evaluation and consideration of early antiviral treatment [16,18].

A prominent epidemiological feature of this outbreak is that the main abnormalities in infected cattle primarily involve the mammary gland and lactation function rather than typical severe respiratory disease [19,20]. Both natural and experimental infection studies have demonstrated substantial viral shedding in milk, with infectious-virus titers in naturally affected cows reported at approximately 10^4^–10^8.8^ TCID_50_/mL, although values vary with disease stage, sampling conditions, and assay method [21]. This feature has important implications for surveillance. Detection and control of H5N1 outbreaks in dairy cattle should not rely solely on traditional influenza surveillance centered on respiratory symptoms. Instead, milk yield, milk quality, milking procedures, and the dairy farm production chain should be central to priority surveillance efforts [16,22].

Although the initial U.S. dairy-cattle outbreak was dominated by genotype B3.13, subsequent detections of genotype D1.1 demonstrated that H5N1 infection in cattle has involved multiple independent avian-to-cattle introductions rather than a single spillover followed exclusively by cattle-to-cattle dissemination. In early 2025, D1.1 viruses were detected in dairy herds in Nevada and Arizona, and whole-genome sequencing showed that the two events were genetically distinct from each other and from the previously circulating B3.13 lineage, supporting separate spillover events [8]. A further D1.1 virus was detected in a Wisconsin dairy herd in December 2025 and clustered closely with viruses circulating in local wild and domestic birds, indicating another independent avian-to-cattle introduction. The D1.1-affected herds in Nevada, Arizona, and Wisconsin generally showed mild or subclinical disease, although the limited number of affected herds precludes firm comparisons with B3.13-associated disease [8,9]. Collectively, these findings demonstrate that dairy-cattle susceptibility is not restricted to genotype B3.13 and highlight repeated introductions from avian reservoirs as an important component of H5N1 outbreak ecology in cattle.

**Figure 1:**
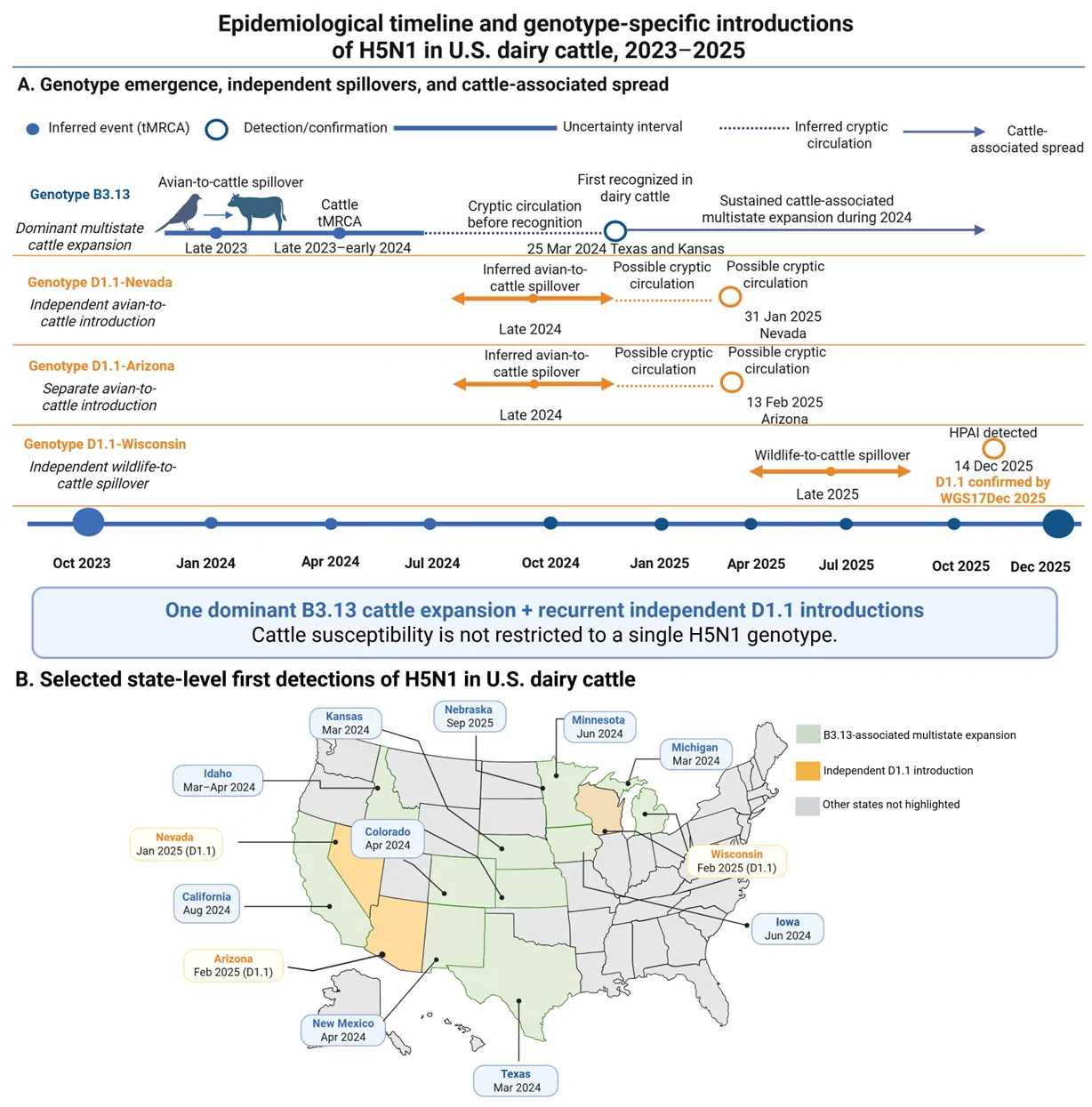
Epidemiological timeline and genotype-specific introductions of H5N1 in U.S. dairy cattle, 2023–2025. (**A**) Schematic reconstruction of the emergence and spread of the major cattle-associated H5N1 genotypes. The dominant B3.13 outbreak is consistent with an avian-to-cattle spillover in late 2023 followed by cryptic circulation and subsequent multistate expansion in cattle. In contrast, genotype D1.1 was introduced independently into dairy cattle in Nevada and Arizona, with an additional distinct wildlife-to-cattle spillover identified in Wisconsin in December 2025. Circles and horizontal bars indicate inferred event estimates and associated temporal uncertainty; detection dates indicate publicly reported confirmation in dairy cattle. (**B**) Selected state-level first detections illustrating the geographic expansion of B3.13-associated infections and the locations of independent D1.1 introductions. Dates and inferred emergence windows are approximate and are based on available epidemiological, genomic, and phylodynamic evidence. Figure created with BioRender.com.

From a One Health perspective, genotype-specific differences may also be relevant to zoonotic risk. A recent study comparing representative D1.1 and B3.13 viruses in human nasal and lower-airway organoid-derived cultures showed that D1.1 replicated to higher titers in several donor-derived cultures and exhibited stronger binding to both α2,3- and α2,6-linked sialic acids than B3.13 [23]. The same study noted that the critically ill or fatal H5N1 cases reported in North America during the study period were associated with genotype D1.1, although the limited number of severe cases precludes attributing increased clinical severity solely to genotype (Figure 1) [23,24]. Therefore, these experimental and epidemiological observations should not be interpreted as evidence that D1.1 is intrinsically more transmissible or pathogenic in cattle or humans, particularly because marked phenotypic variation has been observed among individual D1.1 viruses [9,23]. Notably, the epidemiological behavior of the two genotypes has differed across host populations: B3.13 showed limited persistence in wild birds but sustained expansion in cattle, whereas D1.1 has remained widespread in avian populations but has, to date, shown more limited dissemination in cattle [15]. This contrast suggests that viral fitness is strongly host- and context-dependent rather than an intrinsic property of genotype alone. Consequently, control strategies should address not only onward transmission within and between cattle herds but also continued wildlife-to-cattle spillover through integrated genomic, wildlife, and milk-based surveillance.

## 3. Pathogenesis, Adaptive Evolution, and Virulence Determinants in Dairy Cattle-Associated H5N1

### 3.1. Receptor Binding and Tissue Tropism

Receptor distribution and receptor-binding specificity in dairy cattle-associated H5N1 should be interpreted with consideration of tissue and the glycan-detection reagent or binding assay. Classical avian influenza viruses preferentially recognize α2,3-linked sialylated glycans, whereas human-adapted influenza viruses generally favor α2,6-linked sialylated glycans. However, the bovine mammary gland does not exhibit a simple or mutually exclusive α2,3-versus-α2,6 receptor distribution. Lectin-based studies have demonstrated the presence of both linkage types in bovine mammary tissue, with their abundance and distribution differing among anatomical compartments and according to the lectin used [25,26,27,28]. In particular, Song et al. used Maackia amurensis lectin I (MAL-I), MAL-II, and Sambucus nigra agglutinin (SNA) to characterize bovine pulmonary and mammary tissues. MAL-I preferentially recognizes SAα2-3Galβ1-4GlcNAc structures present in N- and O-linked glycans, whereas MAL-II preferentially recognizes SAα2-3Galβ1-3GalNAc structures in O-linked glycans; SNA is commonly used to detect α2,6-linked sialylated glycans [27]. Their results showed that bovine bronchioles were dominated by α2,3-reactive glycans, whereas lung and conjunctival tissues exhibited predominantly α2,3-reactive staining with a smaller α2,6-reactive component. In contrast, the bovine mammary gland contained substantial signals for both α2,3- and α2,6-linked sialylated glycans [27]. Thus, apparently different α2,3 and α2,6 abundance in bovine tissues may reflect tissue-specific glycan distributions and the distinct glycan structures recognized by individual lectins.

Importantly, lectin staining reflects the distribution of selected sialylated glycan motifs and should not be equated directly with functional receptor usage by a particular influenza virus. This distinction is illustrated by the functional HA-binding experiments of Song et al. The bovine H5 HA examined in that study, corresponding to the HA of A/Texas/37/2024, retained the avian-signature residues Q226 and G228 but contained the T160A substitution that removes the glycosylation site at position 158 [27]. Surface plasmon resonance analysis showed substantially stronger binding of this HA to an α2,3-linked avian receptor analogue than to an α2,6-linked human receptor analogue, although weak but measurable α2,6 binding was detected [27]. Consistent with these findings, the bovine H5 HA bound strongly to bovine mammary gland epithelium, bronchioles, lung, and conjunctiva, whereas human H1N1 and H3N2 HAs exhibited poor binding to the bovine tissues examined [27]. Therefore, the presence of α2,6-reactive glycans in bovine mammary tissue does not imply that the tissue is functionally equivalent to a human α2,6-dominated influenza target or that cattle-associated H5N1 has undergone a complete switch to human-type receptor specificity [29].

Differences among receptor-binding studies may also arise from assay methodology. Song et al. noted that some studies using other experimental systems failed to detect α2,6 binding by bovine H5 HA, whereas their sensitive surface plasmon resonance assay detected weak α2,6 binding only at relatively high HA concentrations [27]. The authors attributed these discrepancies, at least in part, to differences in experimental conditions, HA protein source, glycan presentation, and assay sensitivity. They also acknowledged that their recombinant HA was produced in a baculovirus–insect cell system, in which glycosylation patterns do not completely reproduce those of mammalian cells [27]. Consequently, results obtained from lectin histochemistry, solid-phase glycan-binding assays, glycan arrays, surface plasmon resonance, recombinant-HA tissue staining, and intact-virus experiments should not be considered directly interchangeable. A weak α2,6 signal detected in one assay may therefore be below the detection threshold of another and does not by itself demonstrate a biologically meaningful shift toward human receptor preference.

### 3.2. Polymerase and Internal Gene Adaptation

Adaptive changes in the polymerase complex constitute another key determinant of the enhanced replication capacity of dairy cattle-associated H5N1 viruses in mammalian cells. Among them, PB2-M631L is a prominent molecular marker that emerged following the introduction of genotype B3.13 viruses into dairy cattle populations. This residue is located in a region involved in the interaction between the viral polymerase and host ANP32 proteins, and it enhances the ability of the virus to exploit bovine ANP32, thereby increasing replication efficiency in bovine mammary epithelial cells and other bovine cells [30,31]. PA-K497R has also been identified in early bovine B3.13 isolates, and has been associated with enhanced replication together with PB2-M631L, although its independent contribution requires further functional clarification [22,32]. In addition, newly identified polymerase substitutions, such as PB2-D740N, may also enhance viral replication in mammalian cells, indicating that sustained transmission in dairy cattle provides a novel ecological niche for the continued adaptive evolution of H5N1 [30,33]. Genotype-specific D1.1 introductions further illustrate the diversity of mammalian-adaptive polymerase changes in cattle-associated H5N1 viruses. Whereas PB2-M631L is characteristic of most B3.13 cattle viruses, the Nevada D1.1 viruses carried PB2-D701N, while the Wisconsin D1.1 isolate encoded PB2-E627K together with additional substitutions in HA, PB1-F2, and NS1 [8,9,34]. Notably, despite carrying PB2-E627K, the Wisconsin virus showed lower lethality in mice than previously characterized B3.13 viruses, indicating that individual mammalian-adaptive substitutions do not alone determine viral phenotype and that their effects depend on the broader genetic background [9]. In addition to changes in HA and the polymerase complex, internal genes such as NP and NS1 also contribute to the host adaptation of dairy cattle-associated H5N1 viruses. NP-associated substitutions, including residues linked to BTN3A3 resistance, may assist clade 2.3.4.4b viruses in evading a human anti-avian influenza restriction barrier [31]. Meanwhile, PB2, NP, and NS segments may jointly enhance the capacity of bovine B3.13 viruses to modulate bovine interferon-induced antiviral responses in bovine cells, as indicated by host protein synthesis shutoff, reduced STAT1 phosphorylation, and attenuated interferon-stimulated responses [31]. PB2-E627K, a classical hallmark of mammalian adaptation in avian influenza viruses, was generally absent from early U.S. dairy cattle-associated B3.13 isolates, which instead showed more prominent polymerase-adaptive changes such as PB2-M631L [30]. Notably, PB2-E627K has been detected in human H5N1 isolates associated with dairy cattle exposure, suggesting that bovine-origin H5N1 viruses retain the potential to acquire additional mammalian-adaptive mutations after cross-host infection [35]. Therefore, the adaptive evolution of dairy cattle-associated H5N1 should not be attributed to any single mutation, but rather to the result of multiple factors, including receptor recognition, polymerase activity, escape from host restriction factors, and antagonism of innate immune responses [36].

Even after sustained circulation in cattle, the virus continues to evolve, with mutations such as PB2 E627K and D740N, known to enhance replication in mammalian cells, having been detected in bovine isolates. However, the evolutionary trajectory within cattle appears to be bovine-specific, as the adaptive mutations selected in this host are not entirely congruent with the classical mammalian-adaptive mutations typically observed in other mammals, such as humans. This underscores that post-spillover evolution is a key driver of viral adaptation to novel hosts and of increasing zoonotic concern [34].

The within-host evolutionary dynamics of the virus are also considerably complex. In an experimental study using intramammary inoculation of dairy cattle, the virus was found to replicate rapidly following inoculation and generated multiple intra-host single nucleotide variants (iSNVs), indicating that genetic diversity arises during viral replication within a single host, thereby providing the substrate for subsequent population-level evolution. The study further revealed that viral population composition differed among mammary quarters of infected cows, and that these differences could persist throughout the infection, giving rise to a within-host compartmentalized population structure. Notably, the HA S336N mutation was abundantly present in mammary gland tissues, yet its distribution was uneven across different mammary quarters and tissues, and it remained undetectable in milk samples. Collectively, these experimental findings demonstrate that following infection of dairy cattle, the virus continuously and independently generates novel quasispecies within local microenvironments such as the mammary gland, adapting to changes in the local microenvironment [37].

### 3.3. Mammary Gland Tropism

Mammary gland tropism is a defining feature that distinguishes dairy cattle-associated H5N1 from classical respiratory influenza, and it provides a key basis for understanding the high-titer viral shedding in milk, milking-associated transmission, and mammary gland lesions during this outbreak [33]. Experimental infection studies in cattle offer the most direct evidence for understanding the pathogenic features and transmission dynamics of H5N1 in cattle. Notably, different exposure routes can lead to markedly different infection outcomes. Following oronasal inoculation, the virus can replicate and be transiently shed in the nasal cavity, but clinical signs are mild, and efficient transmission to co-housed sentinel calves has not been observed [33,38]. This suggests that respiratory infection alone is insufficient to fully explain the sustained spread of the virus among dairy cattle during natural outbreaks [12,33]. By contrast, intramammary inoculation of lactating dairy cattle more closely reproduces the core clinical and pathological features observed during natural infection, including fever, a rapid decline in milk production, thickened and discolored milk, and acute necrotizing mastitis [33]. The virus replicates predominantly in inoculated mammary quarters and can be shed into milk at exceptionally high infectious titers. Field studies have reported infectious-virus titers of approximately 10^4^–10^8.8^ TCID_50_/mL in milk from affected cows, whereas controlled intramammary challenge experiments have demonstrated even higher levels under experimental conditions. In a recent dose–response study, quarters inoculated with 10^2^ or 10^3^ TCID_50_ reached >10^12^ TCID_50_/mL in milk by 3 days post-inoculation (dpi), while inoculation with only 10 TCID_50_ still produced titers exceeding 10^11^ TCID_50_/mL [20,39]. By contrast, viral detection in respiratory, fecal, and other non-mammary samples was generally limited.

Ex vivo studies using mammary gland and teat tissues from lactating dairy cattle have further demonstrated the heterogeneous distribution of sialylated glycans across mammary anatomical compartments [25]. Lectin-based analyses detected α2,6-linked sialylated glycans in mammary alveoli, ducts, gland cisterns, and teat cisterns, whereas α2,3-linked glycans showed a more heterogeneous distribution depending on the tissue compartment and the lectin used [25,27,30]. In ex vivo mammary and teat tissues from lactating dairy cattle, dairy cattle-associated H5N1 showed greater replication than chicken-origin H5N1 and human H1N1pdm viruses. At 24 h post-infection, viral titers were significantly higher in gland- and teat-cistern tissues, with the greatest replication observed in teat-cistern explants [27]. In alveolar tissues, dairy cattle-associated H5N1 also replicated, although the differences among viruses were less pronounced than those observed in gland cistern and teat cistern tissues. At 48 h post-infection, dairy cattle-associated H5N1 still maintained high titers in teat cistern tissues, suggesting that the teat cistern epithelium may represent an important local site for efficient viral replication. Immunohistochemistry further revealed that both dairy cattle-associated H5N1 and chicken-origin H5N1 produced clear viral antigen signals in epithelial cells of the gland cistern and teat cistern, whereas human H1N1pdm showed relatively weak antigen staining in these tissues [31,38].

These findings indicate that dairy cattle-associated H5N1 can replicate efficiently in the epithelium of bovine gland cisterns and teat cisterns, providing a histological basis for high-titer viral shedding in milk [40]. However, susceptibility of these tissues following direct exposure should not be equated with evidence that the teat canal is the primary portal of natural infection. The bovine teat canal constitutes an important anatomical and innate barrier to ascending intramammary infection: closure of the teat sphincter and the keratinized lining of the canal restrict entry of microorganisms into the mammary cistern [41]. Experimental removal of teat-canal keratin increases susceptibility to bacterial intramammary infection, whereas mechanical milking can alter teat-end condition and keratin integrity, indicating that barrier disruption or local teat injury may increase susceptibility to ascending infection [42]. Whether these mechanisms similarly facilitate H5N1 entry has not yet been demonstrated. Indeed, experimental H5N1 studies using intramammary infusion bypass this natural barrier; in the dry-cow model of Cool et al., keratin plugs, when present, were deliberately dislodged before inoculum was administered through an udder infusion cannula [37]. Accordingly, the teat canal should currently be considered a plausible portal of ascending H5N1 exposure rather than an established primary route of mammary infection under field conditions.

The anatomical compartmentalization of the bovine udder is also important when considering the kinetics of mammary infection. Each of the four mammary quarters functions as a largely independent gland draining through its own teat. In the dose–response study of Lee et al., separate quarters were inoculated with different doses of H5N1 while one quarter remained uninoculated; despite infectious-virus titers exceeding 10^11^–10^12^ TCID_50_/mL in inoculated quarters, infectious virus was not detected in the control quarter, providing no evidence of inter-quarter viral spread during the observation period [43]. Recent experimental work in dry cows similarly showed that the onset and severity of mastitis remained strongly quarter-dependent and that mock-inoculated quarters were largely unaffected. Compartment-resolved immune profiling further demonstrated predominantly quarter-restricted inflammatory responses, although H5- and N1-specific antibodies became detectable across mammary quarters, indicating that systemic antibody distribution should not be interpreted as evidence of productive inter-quarter viral dissemination [44]. Animal model studies provide further phenotypic evidence that dairy cattle-associated H5N1 has acquired certain features of mammalian adaptation [36,45]. Mouse experiments have shown that bovine B3.13 viruses can establish infection following intranasal and gastric routes and rapidly induce acute disease manifestations, including weight loss, tachypnea, and reduced activity. Compared with some mink-origin, mountain lion-origin, and classical human-origin H5N1 strains, bovine-origin strains can exhibit a more rapid disease course in mice. In C57BL/6J mice, bovine-origin H5N1 replicates efficiently in lung tissue and reaches high viral loads in the brain, accompanied by neurological signs such as tremor, ataxia, circling, and hyperexcitability, suggesting neuroinvasiveness and neurovirulence. Viral antigen was detected in neurons, glial cells, and ependymal cells, together with elevated proinflammatory cytokines [36]. These findings indicate that bovine-origin H5N1 can cause respiratory disease, systemic dissemination, and central nervous system involvement in mammalian models. However, pathogenic outcomes depend on animal models, infectious doses, and exposure routes; therefore, its actual risk to humans needs to be comprehensively assessed with human case data, viral sequence changes, and transmission capacity [45,46].

A recent animal study further demonstrated the distinct mammary tropism of dairy cattle-associated H5N1. Based on the sequence of the U.S. dairy cattle isolate A/dairy cattle/Texas/24-008749-003/2024, the researchers generated a clade 2.3.4.4b genotype B3.13 bovine-origin H5N1 virus using reverse genetics, and compared it with the earlier clade 1 reference strain A/Vietnam/1194/2004. Non-lactating and lactating BALB/c mice were infected. The bovine-origin H5N1 exhibited a higher replication capacity in the mammary glands of lactating mice. At 4 days post-infection, viral titers and M gene copy numbers in mammary tissues were markedly higher than those of the reference strain. Histopathological analysis showed that bovine-origin H5N1 caused obvious structural damage in lactating mammary glands, characterized by shedding and necrosis of alveolar epithelial cells and disruption of glandular architecture. Immunofluorescence showed abundant viral NP antigen in mammary gland epithelial cells, as well as in surrounding adipose and lymphoid tissues. In contrast, the reference strain produced fewer antigen-positive cells in mammary tissue and caused milder tissue damage [47]. These findings further support mammary gland tropism in a lactating mammalian model. Notably, in the mammary glands of non-lactating mice, viral loads and some NP-positive cells could also be detected, but marked glandular epithelial damage was not prominent. This suggests that lactation may provide a more permissive tissue environment for efficient H5N1 replication and pathological injury in the mammary gland. In addition, virus-positive suckling pups were detected among those co-housed with infected lactating dams, with a higher positivity rate in the bovine-origin H5N1 group than in the reference strain group. Virus could be detected in the lungs, brain, or intestine of suckling pups, suggesting that mammary gland infection may be associated with nursing-related exposure and infection risk in offspring [47]. Collectively, these findings indicate that bovine-origin H5N1 not only can replicate in respiratory and systemic tissues, but also displays prominent tropism for the lactating mammary gland, providing experimental evidence to explain the mastitis, high-titer viral shedding in milk, and milk-associated transmission risk during the dairy cattle outbreak [45,47].

Overall, dairy cattle-associated H5N1 viruses have already exhibited multilayered trends of mammalian adaptation. Their HA still predominantly favors avian-type receptor binding, but shows some potential for human-type receptor recognition [27]. Polymerase-associated substitutions, including PB2-M631L and PA-K497R, have been linked to enhanced replication fitness in bovine and mammalian cells [30]. Changes in internal genes such as NP and NS1 may facilitate evasion of host restriction factors and innate immune responses, and animal model studies indicate that some bovine-origin strains possess strong pathogenicity and neuroinvasiveness [31,47]. Although current evidence does not support that bovine-origin H5N1 has acquired efficient human-to-human transmissibility, sustained transmission in dairy cattle may provide opportunities for further accumulation of additional mammalian-adaptive mutations. Therefore, future studies should continue to monitor dynamic changes in receptor-binding profiles, polymerase mutations, immune evasion strategies, and cross-species transmission phenotypes, to enable more accurate assessment of zoonotic risk.

## 4. Clinical Manifestations, Pathological Changes, and Transmission Routes of H5N1 in Dairy Cattle

### 4.1. Manifestations of H5N1 Infection in Dairy Cattle

The clinical manifestations of H5N1 infection in dairy cattle are primarily characterized by abnormal milk production and mammary gland injury, rather than severe respiratory disease. Affected cattle often show reduced feed intake, decreased rumination, depression, fever, mild respiratory signs, or abnormal feces [6]. However, the most indicative signs are a sudden decline in milk yield and changes in milk appearance. Milk may become yellow, colostrum-like, thickened, or coagulated, and some cases are accompanied by evident mastitis. Although most cattle gradually recover within several days to two weeks, milk production may recover more slowly and may take several weeks to return to near pre-infection levels. Elevated mortality reported on some farms is often associated with secondary infections or deterioration of the general condition [5,6].

### 4.2. Pathological Changes in Lung and Mammary

Pathological studies indicate that the main lesions in dairy cattle infected with H5N1 are localized to the mammary gland. Affected cattle may develop acute necrotizing mastitis, characterized by necrosis and exfoliation of mammary epithelial cells, proteinaceous exudates and cellular debris within alveolar lumens, and inflammatory cell infiltration. Viral antigen is mainly localized in mammary alveolar epithelial cells, consistent with the high levels of viral shedding in milk [33,39]. Amy Baker reported that, based on observations up to 24 days post-infection, the pathological damage leading to mammary fibrosis and its long-term impact remains to be determined. The researchers suggested that this infection-associated fibrosis is likely the reason why some dairy cattle, as reported by farmers, fail to regain normal milk production [20]. In contrast, the lungs and other organs typically lack extensive and obvious lesions, indicating that H5N1 infection in dairy cattle manifests primarily as a localized mammary gland infection rather than a systemic disease or severe respiratory infection.

### 4.3. Local Mammary Immune Responses and Protective Immunity

Recent studies have begun to characterize the local and systemic immune responses associated with mammary H5N1 infection. Following intramammary challenge, cytokine and chemokine induction was strongly compartmentalized and largely restricted to infected mammary quarters, with inflammatory responses peaking approximately 3–7 days post-infection. In alveolar compartments, early increases in IFN-γ, TNF-α, CXCL10, CCL2, VEGF-A, and CCL3/CCL4 were followed by induction of IL-1α, IL-1β, IL-6, IL-8, IL-17A, and IL-36RA, consistent with coordinated recruitment and activation of myeloid and lymphoid effector cells [44]. The response also differed between alveolar and teat-cistern compartments, indicating that H5N1-associated mammary inflammation is spatially heterogeneous rather than a uniform udder-wide response.

Evidence for infection-induced protective immunity is also emerging. In experimentally infected lactating cows, serum neutralizing antibodies were detectable from 7 days post-infection and milk neutralizing antibodies from approximately 14 days. When previously unaffected quarters were challenged with the homologous bovine H5N1 virus at 31 days after primary infection, no productive viral replication, milk shedding, or recurrent mastitis was observed, demonstrating short-term protection against homologous reinfection [39]. Longer-term field data further indicate that H5-specific antibodies can persist well beyond clinical recovery: in naturally exposed cattle, 73% retained detectable neutralizing antibodies at 8 months, whereas 50% of the remaining sampled cattle were still positive at 13 months [48]. However, persistence of detectable antibodies should not be equated directly with durable protection, because long-term rechallenge studies and validated correlates of protection in cattle remain unavailable.

### 4.4. Transmission Routes of H5N1 in Cattle Herds

The precise routes responsible for within-herd transmission of H5N1 in dairy cattle remain incompletely understood. The pronounced mammary tropism of H5N1, high concentrations of infectious virus in milk, and persistence of infectious virus on milking-unit surfaces support milk-associated exposure and contaminated equipment as biologically plausible transmission routes [5,20,33,39,49]. Experimental findings from Lee et al. further illustrate this distinction. Direct intramammary inoculation with as little as 10 TCID_50_ established productive infection, with milk titers exceeding 10^11^ TCID_50_/mL, whereas doses of 10^2^–10^3^ TCID_50_ produced titers above 10^12^ TCID_50_/mL [43]. However, these findings demonstrate the availability of infectious virus and the potential for exposure, but do not establish milk or milking equipment as the principal route of natural cow-to-cow transmission. Despite this high mammary susceptibility, sentinel cows repeatedly exposed to contaminated milking claws while co-housed with infected cows remained uninfected during the 14-day exposure period. No spread was also detected from inoculated to uninoculated mammary quarters [43]. These findings demonstrate that efficient mammary replication following direct inoculation does not necessarily translate into efficient natural transmission through contaminated milking equipment.

Field observations likewise support a more complex transmission ecology. Campbell et al. detected infectious H5N1 in milking-parlor air and reclaimed wastewater and observed heterogeneous patterns of quarter-level infection among naturally infected cows [40]. These findings identify additional potential exposure pathways but do not demonstrate efficient airborne- or wastewater-mediated transmission. Raw milk also represents an important source of cross-species exposure and food-safety concern. H5N1-contaminated milk possesses oral infectivity. Mice can develop systemic infection after ingestion, and transmission from lactating dams to pups can occur through nursing [45]. This feature may also explain disease in carnivores such as cats, foxes, and mountain lions following ingestion of contaminated milk or infected animal tissues. In the hospital parlor on more than one dairy farm in Kansas and Texas, cats fed unpasteurized raw colostrum and milk from H5N1-infected dairy cows became infected and developed systemic influenza symptoms, including neurological signs such as depression, ataxia, blindness, and circling [6]. This indicates that the virus can be transmitted to cats through milk, and may also be transmitted to newborn calves via the same route. For humans, properly pasteurized dairy products pose a low risk, whereas consumption or handling of unpasteurized raw milk remains a potential exposure risk.

The 2024 H5N1 outbreak in U.S. dairy cattle revealed that viral transmission within herds differs from the typical respiratory transmission pattern of influenza viruses. Current evidence suggests that milk from infected cattle contains high levels of infectious virus, and that within-herd transmission is more likely associated with milk contamination, mechanical transfer via milking equipment, cross-contamination during milking procedures, and the movement of infected cattle [40]. H5N1 can persist in refrigerated raw milk and on common dairy-contact materials, such as wastewater, stainless steel, polypropylene, and rubber, providing a basis for transmission mediated by milking equipment, milk storage containers, and environmental surfaces [50]. Experimental infection studies have shown that intramammary inoculation can rapidly induce high-titer shedding in milk and mammary gland lesions, whereas oronasal infection of calves produces only limited nasal shedding and does not result in efficient transmission to co-housed sentinel animals [33]. Notably, detection of infectious H5N1 in milking-parlor air and farm wastewater further suggests additional aerosol or environmental exposure routes [40]. Collectively, current evidence supports a multifactorial model involving milk-associated exposure, milking equipment, aerosols, environmental contamination, and other direct or indirect contacts, although the relative contribution of each route under farm conditions remains unresolved.

## 5. Diagnosis, Surveillance, and Control Strategies

### 5.1. Diagnosis of H5N1 Infection in Dairy Cattle

For the H5N1 outbreak in dairy cattle, diagnostic and surveillance strategies should shift from a reactive approach, which tests only after clinical disease is identified, toward an active surveillance system centered on milk samples. Compared with nasal swabs, feces, or general environmental samples, milk samples more directly reflect the key pathological feature of this outbreak, which is efficient viral replication in mammary epithelial cells and massive shedding through milk [51,52]. Therefore, milk testing is not only a cost-effective and efficient means of pathogen detection, but is also more suitable for integration into routine dairy farm workflows, allowing early identification of cattle with abnormal milk production, rapid assessment of the infection range within herds, and regional outbreak early warning. In particular, when respiratory signs are atypical and mortality is low, passive surveillance based primarily on fever, coughing, or mortality may easily underestimate the true scale of mild and subclinical infections.

In terms of testing procedures, rapid antigen tests can serve as on-farm screening tools and are suitable for use in milking parlors, isolation pens, outbreak farms, or before and after animal movement [33]. This method is simple to perform and provides rapid results, enabling rapid identification of suspected infected cattle and immediate isolation. However, its performance is affected by viral load, milk characteristics, sampling time, and sample handling, and it cannot replace nucleic acid testing. Samples that test positive by antigen assay, originate from clinically suspected cattle, or are collected from high-risk herds should be further confirmed by RT-qPCR or digital PCR. Digital PCR may offer higher sensitivity for samples with low viral loads or complex milk matrices and can serve as a supplementary approach for regional surveillance and low-level contamination detection. Dilution of milk samples 1:3 in molecular transport medium prior to RNA extraction provided the best results for dilution of inhibitory substances and a good recovery rate of influenza RNA, reaching approximately 10% to 12.5%. While several tested methods achieved satisfactory detection rates in normal milk with high viral loads, the 1:3 dilution proved most effective in balancing inhibitor dilution with adequate RNA recovery [53,54]. Moreover, standardized protocols have a substantial impact on the sensitivity of H5N1 detection in milk. The U.S. Department of Agriculture’s National Veterinary Services Laboratories (NVSL) have already issued official regulatory protocols for H5N1 testing of milk samples, which include procedures for sample processing and recommendations for commercial extraction and PCR kits [54]. For samples with high viral loads, virus isolation and whole-genome sequencing should be performed whenever possible to clarify viral origin, transmission chains, genotype changes, and the emergence of mutations in PB2, PA, HA, or other genes associated with mammalian adaptation or transmission risk [30,55].

### 5.2. Surveillance of H5N1 Outbreak in Dairy Cattle

The surveillance system should include individual milk testing, bulk tank milk testing, environmental sampling, and antibody surveillance. Individual milk testing is suitable for identifying infected cattle and guiding isolation measures. Bulk tank milk testing is useful for early warning at the farm or regional level. However, a positive result only indicates the possible presence of infection within the herd and cannot directly identify specific infected animals, necessitating follow-up individual sampling. Environmental testing of milking equipment, milk storage containers, wastewater, floors, and surfaces related to human contact can assess the extent of viral contamination and the efficacy of cleaning and disinfection protocols [50]. Milk or serum antibody testing can complement pathogen detection by providing information on prior infection, herd immune status, reinfection risk, and immunity derived from infection or vaccination [39]. These different testing approaches are not interchangeable, but should be combined according to the purpose of surveillance. Rapid screening relies on antigen testing or qPCR, outbreak tracing relies on sequencing, herd immunity assessment relies on antibody testing, and environmental risk assessment relies on combined monitoring of milk and farm environmental samples.

Control measures should focus on viral shedding in milk and the milking process. Since milk from infected cattle may contain high levels of infectious virus, the virus can remain infectious for a certain period in refrigerated milk and on materials such as stainless steel, plastic, and rubber, and the milking process may become a key node for within-herd transmission and environmental dissemination. Milk system data (such as real-time milk yield and electrical conductivity per cow) should be used for preliminary early warning to flag abnormal animals [56,57]. Flagged cows should then be subjected to on-site rapid antigen testing, and those testing positive should proceed to the isolation workflow. Suspected cows may be held in temporary isolation pens within the barn while awaiting test results. Confirmed cases should be moved to a dedicated sick cow barn located downwind. For confirmed cows, milking should be performed directly in the isolation barn using a mobile milking unit, completely avoiding entry into the main milking parlor. Milk from confirmed cows should be discarded, and the discarded milk should be inactivated (e.g., by heat treatment) before disposal [51]. In addition to the milking process, farms should manage animal, personnel, and logistics factors that may contribute to viral spread. Affected farms should reduce or suspend non-essential cattle movements, trace recently introduced or transferred cattle, and assess risks associated with shared equipment, transport vehicles, and personnel movement. Entry of wild birds, cats, rodents, and other wildlife into barns and milking areas may increase the risk of cross-species transmission and viral spillback. Therefore, management of feed, water sources, discarded milk, and animal carcasses should be strengthened [6]. In particular, feeding raw milk or abnormal milk to cats, calves, or other farm animals should be prohibited to prevent the establishment of new infection chains through foodborne exposure. Overall, control of H5N1 in dairy cattle should not be limited to isolating diseased cattle after detection, but should integrate milk testing, milking process management, environmental disinfection, cattle movement tracing, and dairy supply chain oversight into a coordinated, farm-to-supply-chain control program.

### 5.3. Vaccination Strategies and Implementation Considerations

Vaccination may provide an additional tool for controlling H5N1 in cattle. An inactivated rabies virus-vectored H5 vaccine was well tolerated in heifer calves and induced neutralizing antibodies against both clade 1 and contemporary clade 2.3.4.4b H5 viruses [58]. More recently, an adenoviral vaccine expressing a centralized-consensus H5 antigen induced serum IgG, nasal IgA, and H5-specific T-cell responses in Holstein calves [59]. However, neither study included infectious H5N1 challenge in cattle. Several additional vaccine platforms have shown protective efficacy in preclinical models. A replicating RNA vaccine encoding a contemporary bovine clade 2.3.4.4b HA provided complete protection against homologous lethal challenge in mice, whereas a historical clade 1 HA provided only partial protection [60]. DNA and mRNA-LNP vaccines based on contemporary H5N1 antigens also protected mice against lethal challenge [61,62], while NS1-deficient live-attenuated vaccines induced systemic and mucosal immunity and protected mice or hamsters [63,64]. Thus, current cattle studies demonstrate immunogenicity but do not yet establish protection against mammary infection, viral shedding in milk, or transmission. Representative vaccine platforms are summarized in Table 1.

An important unresolved issue is the breadth of cross-protection across antigenically and genetically distinct H5N1 viruses. Available findings are not entirely uniform across vaccine platforms and experimental models. In cattle, the clade 1 RABV-H5 vaccine elicited serum antibodies capable of neutralizing a contemporary clade 2.3.4.4b cattle H5N1 virus, suggesting some degree of cross-clade humoral immunity [58]. The adenoviral H5CC vaccine likewise elicited broad binding and cellular responses in calves, but these responses have not yet been linked to protection against infectious challenge in cattle [59]. In contrast, the repRNA study in mice showed that immunity elicited by the historical A/Viet Nam/1203/2004 HA provided substantially weaker protection against contemporary bovine H5N1 than a matched clade 2.3.4.4b HA [60]. Natural H5N1 infection in dairy herds also produces an antibody response dominated by clade 2.3.4.4b HA, with weaker cross-reactivity to more divergent H5 antigens [65]. Together, these findings indicate that cross-reactive antibody binding or neutralization should not automatically be equated with equivalent protection across H5 lineages. Continued antigenic characterization will therefore be required to determine whether candidate vaccines remain protective against newly emerging cattle viruses, including genetically distinct D1.1 introductions, and whether vaccine antigens require periodic updating.

Practical implementation will require integration of vaccination with existing surveillance and biosecurity programs. Because H5 antibodies in serum or milk may result from either infection or vaccination, serological surveillance should be supported by virological testing, vaccination records, and, where feasible, DIVA strategies. Before routine use in dairy herds, further studies are needed to determine efficacy in lactating cows, duration and breadth of protection, effects on milk shedding and transmission, and feasibility of large-scale deployment. Vaccination should therefore complement, rather than replace, surveillance, biosecurity, movement control, and measures aimed at reducing repeated avian-to-cattle spillover.

## 6. Public Health Significance, Challenges, and Future Directions

The public health significance of the 2024 H5N1 outbreak in U.S. dairy cattle lies in the shift in H5N1 risk from the traditional wild bird–poultry interface to the dairy production system. Compared with outbreaks in wild birds or poultry, dairy farms present a distinct risk profile. On the one hand, lactating dairy cattle can shed large quantities of virus through milk, making raw milk, milking equipment, and the production environment potential sources of contamination [40]. On the other hand, occupational exposure represents an important public health concern. Milkers, veterinarians, transport personnel, and raw-milk handlers frequently come into contact with infected animals, milk, and contaminated farm environments, creating opportunities for exposure of the conjunctival and respiratory mucosa [66,67]. Although direct milk splashes to the eyes remain an important recognized exposure pathway, airborne exposure within milking parlors should also be considered. Recent field surveillance detected H5N1 viral RNA in dairy-farm air and recovered infectious virus from air samples collected in milking parlors during milking, while particle measurements indicated the generation of both submicron and larger aerosols [40,68]. Milking activities, including forestripping and handling of residual contaminated milk, may contribute to aerosol generation. In addition, high-pressure cleaning of contaminated parlor surfaces or waste materials could theoretically resuspend or aerosolize virus-containing droplets and organic material, although H5N1-specific aerosol generation during parlor sanitation has not yet been directly quantified. Agricultural studies with other biological contaminants have shown that high-pressure washing can forcibly aerosolize material deposited on surfaces and substantially increase worker aerosol exposure, supporting caution when this practice is used in contaminated environments [69]. Accordingly, workers handling diseased cattle, abnormal milk, contaminated equipment or wastewater, and those cleaning affected milking parlors should use appropriate eye and respiratory protection and minimize procedures that generate unnecessary splashing or aerosols [22].

The main challenge in current control efforts is not the lack of diagnostic tools, but the absence of a closed-loop data system connecting farms, diagnostic laboratories, genomic surveillance platforms, and public health systems [16]. The atypical clinical signs of infection in dairy cattle increase the risk of missed detection under passive surveillance. Although milk samples are suitable as core surveillance specimens, the sensitivity, specificity, and appropriate application settings of different testing methods in complex milk matrices still require standardization [52]. Bulk tank milk testing offers early-warning value, but it cannot directly identify infected individuals. Incomplete information on cattle movements, shared equipment, transport vehicles, and personnel movement further complicates the reconstruction of transmission chains. Moreover, without metadata, such as sampling time, location, host source, clinical presentation, and farm management information, viral sequences alone are difficult to interpret in terms of the true epidemiological significance of specific mutations [16,55].

Future work should focus on three priority areas under a One Health framework. First, standardized surveillance procedures for H5N1 in dairy cattle should be established, with clearly defined optimal applications for individual-cow milk samples, bulk tank milk, environmental samples, serum, and human samples. Second, whole-genome sequencing results should be integrated with cattle movement records, farm spatial information, milking procedures, environmental testing results, and occupational exposure data to assess the relative contributions of milk-mediated transmission, equipment-mediated transmission, animal movement, and cross-species exposure. Third, the practical effectiveness of isolation of diseased-cattle, last milking strategies, equipment disinfection, abnormal milk disposal, occupational protection, movement restrictions, and vaccination strategies should be systematically evaluated. Overall, mammary gland tropism, high-titer viral shedding in milk, and milking-related exposure constitute the core features that distinguish this outbreak from previous avian outbreaks and sporadic mammalian infections. These features also indicate that H5N1 risk assessment needs to shift from single-outbreak response toward long-term early warning and systematic risk control.

## 7. Conclusions

Since the first confirmation of H5N1 highly pathogenic avian influenza in U.S. dairy cattle in March 2024, the transmission patterns and pathogenic characteristics of the outbreak have revealed epidemiological and pathogenic features that differ substantially from those typically associated with avian influenza in birds. In contrast to the conventional respiratory tropism of influenza viruses, H5N1 targets the mammary gland as its primary organ in dairy cattle. The virus replicates efficiently in mammary epithelial cells and is shed in large quantities through milk, with shedding titers reaching up to 10^4^ to 10^8.8^ TCID_50_ per milliliter. Current evidence suggests that the virus may spread within herds primarily via an “oral–mammary” route, potentially through calf suckling or cross-suckling behavior among adult cattle. In addition, contaminated milking equipment, personnel operations, and unpasteurized raw milk may serve as critical vehicles for viral dissemination within and between farms. Farm workers in direct contact with infected cattle face a clear risk of occupational exposure; although most cases present with mild symptoms such as conjunctivitis, there is no evidence to date of sustained human-to-human transmission. At the prevention and control level, priority should be given to milk sample screening, isolation of suspected animals, and pasteurization of milk products, so as to effectively protect the health of workers and ensure dairy product safety. These measures represent not only the immediate focus of outbreak response but also a vital component of a broader One Health strategy.

## Figures and Tables

**Table 1:** Experimental H5N1 vaccine candidates relevant to the control of infection in dairy cattle.

Vaccine Platform	Antigen/Strain	Model	Immunization	Main Findings	Cross-Protection	Major Limitation for Cattle Application
Inactivated rabies virus-vectored H5 (RABV-H5)	H5 HA from A/Viet Nam/1203/2004, clade 1	Cattle (Angus heifers)	SC; prime or prime–boost ± adjuvant	Well tolerated; induced H5 neutralizing antibodies; adjuvanted prime–boost gave the strongest response	Neutralized contemporary clade 2.3.4.4b A/cattle/Texas/56283/2024	Beef rather than lactating dairy cattle; no H5N1 challenge; effect on milk shedding/transmission unknown; cattle correlate of protection undefined [58]
Adenoviral H5CC, IM + IN	Consensus H5 HA (H5CC); Ad5/Ad6 or Ad28/Ad48 vectors	Holstein dairy calves	Holstein calves; IM + IN heterologous prime–boost (days 0 and 28)	Broad serum IgG, nasal IgA and H5-specific T-cell responses	Cross-reactive systemic, mucosal, and T-cell responses to diverse H5 antigens; limited neutralization of contemporary 2.3.4.4b viruses; no cattle challenge. Complete protection against divergent H5N1 challenge, including bovine H5N1, demonstrated only in mice.	No cattle challenge; limited neutralization of contemporary bovine H5N1 [59]
Replicating RNA (repRNA)	HA from contemporary A/bovine/OH/B24OSU-342/2024	Mouse	Single IM dose	Matched bovine HA vaccine completely protected against lethal homologous challenge	Historical A/Viet Nam HA produced only partial protection, emphasizing antigen matching	No cattle data; mouse challenge phenotype may not reflect dairy-cattle infection [60]
DNA and mRNA-LNP	HA + NA from A/Texas/37/2024	Mouse	IM prime–boost	Strong HA/NA immune responses and complete protection against lethal challenge	Contemporary 2.3.4.4b antigen; broader genotype protection not established	No cattle efficacy or milk-shedding data [61]
H5HA mRNA-LNP (DS8390)	HA from clade 2.3.4.4b A/chicken/Ghana/2021	Mouse	IM prime–boost	Protected against lethal dairy-cattle H5N1 challenge and markedly reduced tissue viral load	Demonstrated protection against a heterologous cattle clade 2.3.4.4b virus	No cattle data; breadth against other cattle genotypes, including D1.1, unknown [62]
NS1-deficient live-attenuated H5N1	Cattle-origin A/Texas/37/2024-derived antigen/background	Mouse	Single intranasal dose	Attenuated and immunogenic; protected against lethal wild-type H5N1 challenge	Contemporary cattle-origin antigen	No cattle testing; durability requires further study; live-vaccine reassortment and shedding require evaluation [63]
DelNS1 H5N1 live-attenuated vector	H5/NA based on cattle or mink clade 2.3.4.4b viruses	Mouse and hamster	Single intranasal dose	Induced neutralizing antibodies, mucosal IgA and T-cell responses; protected against lethal challenge	Protection against cattle/mink H5N1 variants in small animals	No cattle studies; field safety, shedding and transmission effects unknown [64]

## Data Availability

No new data were created or analyzed in this study. Data sharing is not applicable to this article.
